# The role of embedded research in quality improvement: a narrative review

**DOI:** 10.1136/bmjqs-2015-004877

**Published:** 2016-04-29

**Authors:** Cecilia Vindrola-Padros, Tom Pape, Martin Utley, Naomi J Fulop

**Affiliations:** 1 Department of Applied Health Research, University College London, London, UK; 2 University College London Hospitals NHS Foundation Trust, London, UK; 3 Clinical Operational Research Unit, University College London, London, UK

**Keywords:** Healthcare quality improvement, Health services research, Quality improvement

## Introduction

The use of research evidence to facilitate improvements in healthcare quality continues to be a topic widely debated by scholars and practitioners.^1^ ^2^ The concept of ‘knowledge mobilisation’ has been developed, with strategies to help bridge this gap.^3^ These strategies include the development of “a culture of partnership between academic researchers and decision-makers to assist in strengthening the development of policy, practice and social innovation, or the co-production of knowledge”.^3^ ^4^ It is based on the premise that knowledge that is collected and created ‘on the ground’, through daily interaction and negotiation with practitioners, managers and service users,^4^ will provide better insight into the issues affecting these stakeholders, be more relevant to the local context and will, therefore, be more easily incorporated into changes in practice.^5–11^

Different strategies have been used internationally to promote knowledge coproduction.^12^ Several of these strategies entail the creation of partnerships between academic and healthcare organisations.^13–19^ In some cases, these partnerships use ‘boundary spanners’,^19^ ^20^ ‘knowledge brokers’^21^ or other intermediary roles,^5^ where individuals work to link practitioners with knowledge and develop organisational capacity to carry out and incorporate research into practice.^18^ ^22^ One type of intermediary role is the embedded researcher. There are multiple definitions of embedded research and one of the goals of this review is to explore the wide range of meanings associated with this term. However, as a starting point, we used the definition proposed by McGinity and Salokangas,^23^ where embedded researchers are defined as those who work inside host organisations as members of staff, while also maintaining an affiliation with an academic institution. Their task is seen as collaborating with teams within the organisation to identify, design and conduct research studies and share findings which respond to the needs of the organisation, and accord with the organisation's unique context and culture.^23^ The role of embedded researchers differs from that of knowledge brokers and boundary spanners. Embedded researchers may use techniques used by knowledge brokers such as knowledge management, linkage and exchange and capacity building (based on the definition of knowledge broker used by^5^ ^6^ ^21^). Furthermore, they might operate as ‘boundary spanners’ in the sense that they work across organisational boundaries.^5^ However, their main purpose is to carry out research, to coproduce knowledge. The research is therefore produced through a collaborative and participative process, and it is jointly ‘owned’.^23^

The use of embedded researchers within and outside of healthcare appears to be a growing practice,^14^ but, to our knowledge, there are no published reviews of the characteristics of this approach, its potential role and the challenges it might face. Therefore, this review addresses the following questions. What are the characteristics of embedded research? What is the potential role for embedded researchers to facilitate improvement research that makes a difference? What are the challenges of such models? How can the lessons learned by embedded researchers in other sectors be applied to embedded research in healthcare? Our review synthesises the available literature on the experiences of researchers using the embedded research approach and presents a series of lessons learned for its application to research aimed at quality improvement in healthcare.

## Methods

We carried out a narrative review^24–26^ to explore the role that embedded researchers could play in improvement efforts in healthcare. We conducted a two-stage bibliographic search of publications in English from 1937 to November 2015 using MEDLINE, Web of Science, PsychInfo, ProQuest Social Science and CINAHL Plus. In the first stage, we used the following search terms: ‘embedded research’ OR ‘embedded researcher’ OR ‘embedded researchers’ OR ‘researcher-in-residence’ OR ‘researcher in residence’ OR ‘boundary spanners’ OR ‘boundary spanner’. We included ‘boundary spanner’ as a search term to account for the work of researchers working across multiple organisations as this was an important aspect of the definition of embedded research we used as a starting point.^23^ Due to the contested nature of the definition of embedded research, we carried out a second stage of the search based on the identification of terms used in the articles that we included in the first stage of the review. These terms were: ‘intermediaries’ OR ‘transient government officials’ OR ‘embedded scientist’ OR ‘engaged scholar’ OR ‘knowledge broker’. In the same way, we iterated our search terms in this two-stage process, we also applied the working definition of embedded research outlined above flexibly in order to capture the wide range of approaches being employed in this emerging field (see under ‘Defining embedded research’ in Results section).

We conducted a review of bibliographies to identify further relevant publications and hand-searched the following journals: *BMJ*, *BMJ Quality and Safety*, *Anthropology in Action*, *BMC Health Services Research* and *Implementation Science*. These journals were selected based on our findings of the initial searches. Results were combined into RefWorks, and duplicates were removed.

The inclusion criteria were peer-reviewed journal articles focused on the embedded research approach both within and outside healthcare. The latter was included because the reflections on the process of carrying out research as part of an organisation in other sectors may be valuable for healthcare research. We excluded publications that were published in languages other than English.

**Figure 1:**
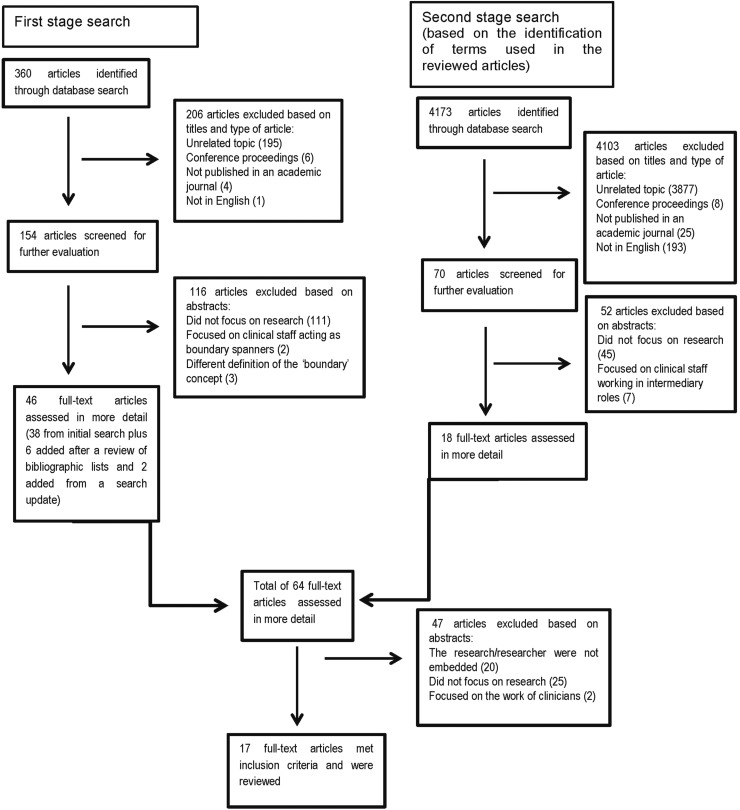
Flow diagram of publication selection process.

The exclusion criteria are presented in figure 1. The included articles were analysed using a data extraction form developed in RedCap (Research Electronic Data Capture), which was created after the initial screening of full-text^27^ articles (see online supplementary appendix 1). We analysed the content of the articles in relation to the questions set out above. In addition, we captured themes emerging from the articles to include relevant issues not covered by our initial research questions.

10.1136/bmjqs-2015-004877.supp1 Supplementary appendix

## Results

The first search yielded 360 published articles (see figure 1). The articles were screened in RefWorks by two of the authors (CV-P and TP) based on title, language, general topic, and type of publication, resulting in a total of 154 articles. Screening based on abstracts resulted in 38 articles for full-text review. The review of bibliographies yielded six more articles. The second search yielded 4173 articles; initial screening based on title led to 70 articles. Screening based on abstracts resulted in 20 articles for full-text review (see figure 1). The number of articles included for full-text review from both search stages was 64. After the full-text review, 47 articles were excluded. Seventeen articles were included in the final selection.

Our review of the literature indicates that the embedded research approach is an emerging trend within and beyond healthcare. Table 1 contains a description of the publications included in the review. The review contains eight healthcare-related publications and nine non-healthcare-related publications.

**Table 1:** Characteristics of the articles included in the review and definition of embedded research/researcher

Article	Country	Year	Sector	Definition of embedded research/researcher	Characteristics of the embedded researcher/researchers	Perceived benefits	Perceived challenges
Groundwater-Smith and Mockler^28^	Australia	2002	Education	“Researcher in Residence is a phrase used to connote a role analogous to a ‘writer in residence’ or ‘artist in residence’. However, it is a positioning which is distinctive from being a resident in the fullest sense of the term, in that the researcher maintains her affiliation with her university and broader educational research and professional development consultancy”.^28^	Researcher has relative autonomy in the host organisation.	If the embedded position is funded by the host organisation, it allows the researcher to develop long-term relationships with staff and guarantee the sustainability of the research.	The researcher might face difficulties understanding and navigating the terrain of the host organisation, and thus be seen permanently as an ‘outsider.’
Reiter-Theil^29^	Switzerland	2004	Health	Researchers carrying out research in the relevant context without performing the same practices as those studied.	Carried out observations and documented clinical practice. Acted as ‘some kind of team member.’	Provided valid and meaningful results to practice. Allowed researchers to ask the ‘right’ questions.	Effort required to maintain trust, reliability and stamina among clinicians and research team.
Hentschel *et al*^30^	Germany/Switzerland	2006	Health	“Method of the embedded researcher […] allows for a combination of methods of systematic observation and case documentation in a naturalistic setting”.^30^	Carried out observations and documented clinical practice. Used the embedded research method outlined in.^29^	Not specified	Not specified
Pyett *et al*^31^	Australia	2008	Health	Three researchers-in-residence employed by an aboriginal community-controlled health organisation. Two of the researchers are also affiliated to a university.^31^	–	–	–
Nutley *et al*^32^	UK	2008	Education	“Interactive relationship between researchers, managers, and practitioners in the development of research-based guidance, protocols, and tools”.^32^	Based on the translation of research into practice by individuals in local policy or management roles. Research becomes embedded in systems, processes and standards.	Model may be suited to staff in certain circumstances and where practice tools can be tailored to the local context.	Embedded research model needs to adapt to the particular research questions that need to be addressed.
Jenness^33^	USA	2008	Judicial system	“I use the term embedded researcher to talk about something […] that provides multiple vantage points from which to view the scene: occupying multiple locations within and under the control of a single field of play while also moving from one site to another, one level of analysis to another, and one constituency to another-ultimately having a presence as a didactic participant throughout a field of play”.^33^	Worked as ‘a public servant.’ Carried out in-depth fieldwork with inmates and wardens in the California prison system.	A good way to get a unique perspective, insight and data.	The researcher has relations of dependence with the host organisation, which might shape the type of research that is conducted and the dissemination of findings.
Wong^34^	Australia	2009	Education	Researcher employed full-time to conduct research within an organisation.	Worked alongside, shared office space and socialised with practitioners. Embedded researchers participated in six studies focused on programme evaluations (outcomes and processes) and research projects addressing questions that arose from practice and/or the literature. One of the goals was also to increase staff engagement with research.	Increased local staff members' capacity to conduct research. Contributed to the continuous improvement of programme delivery. Provided local staff the opportunity to reflect on their work, increase their skills and knowledge, and collaborate with other staff members. Increased the organisation's capacity to inform policy and practice.	Not all researchers are suitable for embedded roles; personal characteristics and dispositions play an important role. There needs to be a ‘good fit’ between the researcher and the organisation. Sharing of findings might be restricted if the organisation owns the intellectual property.
Nutley *et al*^35^	UK	2009	Social care	“Research enters practice indirectly; it becomes embedded in systems, processes and standards. […] Research knowledge is translated into frontline practice activities by intermediaries”.^35^	Translation of research-insights into practice activities.	Model may be suited to staff in certain circumstances and where practice tools can be tailored to the local context.	Embedded research model needs to adapt to the particular research questions that need to be addressed.
Hackett and Rhoten^36^	USA	2011	Science policy	Two researchers worked as transient government officials at the National Science Foundation (NSF) “with responsibility to manage a research program, direct a division, develop new research solicitations, serve on NSF policy committees, and conduct our research”.^36^	Managed a research programme and carried out research. Developed new research solicitations. Served on internal committees.	Guarantees the researchers’ access to staff members and provides the opportunity for witnessing internal events and processes. Allows the researchers to engage in discussion and reflection with members of the organisation. Allows researchers to engage with real problems in a real context.	Researchers might face restrictions in the dissemination of findings. Researchers occupy an intermediate status with commitments to often conflicting values.
Lewis and Russell^37^	UK	2011	Health	“A situationally appropriate way of ‘doing ethnography’ that is founded on the principles and practice of immersion fieldwork while being responsive to working with reflexive collaborators, adaptive to the requirements of ethics and other forms of research regulation, and accommodating to audiences eager for new forms of ethnographic output”.^37^	Researcher acts as ‘some kind of team member.’ Researcher maintains a collaborative relationship with coworkers. Researcher uses traditional principles of ethnographic fieldwork.	Enables researchers to respond to collaborators’ needs and expectations.	Requires a certain critical distance. Researchers must deal with working in a state of 'in-between-ness.’
Rowley^38^	UK	2014	Education	“Individuals or teams who are either university-based or employed undertaking explicit research roles within host schools or other educational organizations, legitimated by staff status or membership with the purpose of identifying and implementing a collaborative research agenda”.^23^	Carried out quantitative and qualitative research to inform future practice. Performed informal tasks to develop trusting relationships. Attended and participated in steering board meetings. Developed reports sharing research findings.	The researcher has access to a wide range of people and informal practices, increasing the depth and diversity of collected data. The research can respond in an ad hoc way to data collection opportunities. Insider knowledge allows the researcher to tailor the research to meet the needs of the organisation.	Ethical regulation procedures cannot always be adapted to the realities and timeframes of embedded research. The researcher establishes commitments with multiple subgroups within the organisation which can sometimes come into conflict. The researcher operates in a state of ‘in-between-ness’ between the organisation and university.
Marshall *et al*^14^	UK	2014	Health	Researcher is a core member of the delivery team, with a sense of shared responsibility for the success or failure of an improvement initiative.	Researcher establishes trusting relationships with staff. Researcher considers their expertise to be complementary to that of other team members.	The embedded research approach addresses the barriers between researchers and practitioners, leading to the negotiation of knowledge and increasing the chances it will be used in practice.	Embedded researchers are subjected to different requirements for career development in the academic and health organisations, which are not always compatible.Research findings might conflict with organisational goals. Further development and evaluation of the approach are required.
Marshall^3^	UK	2014	Health	“Researchers-in-Residence blur the traditional boundary between their expertise and that of the health service team by becoming an integral part of the team rather than central commentators”.^3^	The researchers are in close connection to routine practice and produce transferrable knowledge.	The researcher brought unique expertise to the team and created new evidence in collaborative form.	Embedded research might not be considered valuable under the reward systems used in most academic institutions. Embedded approaches might put scientific objectivity at risk.
Marshall^39^	UK	2014	Health	“An integrated member of a service-based improvement team”.^38^	Negotiate their knowledge and integrate it with the expertise of practitioners. Researcher interprets research evidence in relation to the local context. Evaluates improvement efforts looking at the intended and unintended consequences of interventions.	Encourages researchers to be more useful to practitioners. Encourages practitioners to be responsive to scientific evidence. Can help deliver better care with limited resources.	There are no set guidelines on the required personal skills and level of experience of the researcher. Embedded researchers must negotiate between their sense of ownership over the work and their independent evaluation.
Duggan^40^	UK	2014	Education	“Individuals or teams who are either university-based or employed undertaking explicit research roles within host schools or other educational organizations, legitimated by staff status or membership with the purpose of identifying and implementing a collaborative research agenda”.^23^	Carried out qualitative research to collect evidence for a new initiative. Devised an evaluation framework. Contributed to funding applications for internal projects.	Allowed the researcher to gain insight into daily practice and the nuances of collaborative work.	Embedded research can be disrupted by policy, personnel or organisational change.
McGinity and Salokangas^23^	UK	2014	Education	“Individuals or teams who are either university-based or employed undertaking explicit research roles within host schools or other educational organizations, legitimated by staff status or membership with the purpose of identifying and implementing a collaborative research agenda”.^23^	Researchers ‘get under the skin’ of organisations in order to document multiple perspectives and processes. A ‘mutually beneficial relationship’ is created between the host organisation and university.	The researcher obtains greater access to the organisation, which facilitates data collection and can help with funding. The host organisation gains academic knowledge and critical approaches to inform its policies and practices.	Embedded research is a complex practice, influenced by organisational pressures, interests, and changes. Funding arrangements create particular power relations and shape the role of the embedded researcher. Flexibility, adaptation and reflexivity are required.
Eyre *et al*^41^	UK	2015	Health	“An emerging model of participative research […] that embraces the concept of ‘cocreating’ knowledge between researchers and practitioners.”^40^	Researcher is embedded in a programme team (employed by university but maintains an affiliation with the health organisations). Based on collaboration, reflection and collective inquiry. The researcher focuses on initiating change through shared learning and knowledge of the local context.	Increases research impact.	The embedded research approach needs to respond to the complex processes and structures of the organisations where the researcher works.

### Defining embedded research

There is currently a wide spectrum of research activities that share characteristics that embody ‘embedded research’. While our working definition included a requirement that embedded researchers need to have dual affiliation (to an academic institution and the host organisation),^23^ we found that five of the articles included in the review did not discuss issues of dual affiliation. These articles, however, satisfied all of the other characteristics outlined in our working definition. One additional article clearly stated that the researchers were not affiliated to an academic institution while they were embedded. We included this article because the authors reflect on the positive and negative aspects of not having this dual affiliation. The approach we envision as embedded research is still in early stages of development, but despite variations in the affiliation of researchers, a series of common features can be identified (see box 1).

Box 1 **Characteristic features of embedded research**

1. Researcher is usually affiliated to an academic institution as well as an organisation outside of academia, thus working in a state of ‘in-between-ness’.
2. Researcher develops relationships with staff and is seen as part of the team.
3. Researcher generates knowledge in conjunction with local teams (coproduced) which responds to the needs of the host organisation.
4. Researcher builds research capacity in the host organisation.

#### Becoming part of the organisation

Lewis and Russell^37^ regard it as essential that the researcher undergoes a process of immersion within his or her host organisation. By ‘being there,’ the researcher is able to grasp the challenges faced by the organisation, its goals and interests and the contexts where these play out.^23^ ^36^ ^37^

One of the main challenges of improving the quality of healthcare is the development of an organisational culture that is supportive and committed to improvement.^1^ As Dixon-Woods *et al*^1^ have argued, “problems can occur when improvement efforts run counter to centrally driven national pushes and pressures or are introduced into environments already suffering organisational stress from mandated requirements”. By being immersed in the organisation, the embedded researcher can gain greater understanding of the pressures and problems faced at different levels of the organisation and tailor improvement strategies accordingly.^14^ ^23^

#### Developing relationships with staff

Physical presence alone is not enough to become an embedded researcher. As Wong^34^ highlights, an important component of ‘embeddedness’ lies in the quality and types of relationships the researchers foster with staff. Through these relationships, the researchers gain trust and are seen as members of the team.^29^ ^34^ ^39^ Their positionality, or the way researchers see themselves and are seen by others in the organisation, varies in relation to the people involved and the context.^37^ In his embedded work, Duggan^39^ established different collaborative relationships, such as: ‘critical friendship’ (working in equal relation to the project manager), ‘critical nephewship’ (working in a junior position) and ‘critical orphanship’ (unattached to the project team). These relationships allowed him to reach out and capture the views of staff at different levels of management, acting as an equal with some staff and in a more subordinate position with others. When acting as an ‘unattached’ researcher, he had more flexibility to participate in new activities within the organisation.

Establishing collaborative relationships with local teams is important for uncovering the different viewpoints of staff members regarding the issues faced by the organisation and how they could be addressed. This insight into the wide range of perspectives coexisting in the organisation can allow the researcher to make research findings more relevant to local end users, promote ownership of these findings and anticipate potential sources of tension produced by competing views.^38^ Improvement initiatives in healthcare frequently (but by no means always) emerge from the interests of senior groups within organisations who have particular ideas about the problems faced by the organisation, their causes and the best ways to solve them. These initiatives are often developed without the involvement of those who will experience changes in their daily practice and are imposed as a top-down measure.^42^ As a consequence, they often fail.^1^ The spread of innovations can also encounter similar obstacles when it is meant to intersect different groups and cross professional boundaries.^43^

Embedded researchers seek to tackle this issue of top-down approaches by considering the fact that each organisation has multiple subgroups with their own views of how the organisation works and how services should be organised.^36^ ^37^ By working with these groups on an ongoing basis, embedded researchers are able to understand the complexity of the situations faced by the organisation and propose strategies that respond to the interests of a wider range of stakeholders.^38^

#### Critical reflection by the researcher and local team

Some authors argue that the creation of these collaborative relationships can be enriched when the researcher employs a reflexive approach.^23^ ^37^ ^38^ Reflexivity entails a conscious exercise of thinking about the position the researcher occupies as an individual, and as part of the organisational context.^37^ Reflexivity helps the researcher maintain a clearer idea of their role and capacity to intervene.^38^ It also supports a continuous reassessment and adjustment of the researcher's practice.^34^ When researchers are able to foster individual reflexivity, they become aware of potential barriers to the research process, and can thus adapt research activities to address the needs and interests of all involved parties, and create stronger relationships with the people participating in the research project.^37^ ^34^ Furthermore, when shared with other members of the team in the form of collective reflexivity, this exercise provides a way of fostering critical thinking within the team even after the researcher leaves the organisation—a form of capacity building.^34^

### Informing practice

Some have argued that in the traditional model of research, there is a disconnection between ‘producers’ and ‘consumers’ of research evidence.^3^ ^44^ ^45^ As a consequence, organisational decision-making is not always informed by health service research evidence.^3^ One of the goals of embedded research is the rapid delivery of research findings and their quicker incorporation into improvements in practice.^14^ Due to their immersion within the organisation, embedded researchers can produce research that is more relevant to the ‘end user’ and can give advice and flag issues in formal and informal ways.^37^ ^34^

Having regular meetings with clinical teams and management groups to discuss progress of their work is proposed as a useful mechanism for the provision of iterative feedback.^34^ Such meetings are about discussing the research progress and maintaining relationships, and also about ensuring that all relevant members of the host organisation still feel they ‘own’ the problem, and will be willing to own the solution, too. The process of engaging staff to own the problem and support service improvement has been widely discussed in the healthcare quality improvement literature. Dixon-Woods and colleagues have argued that ‘soft’ and ‘hard’ tools might be needed to persuade staff to change current practices in healthcare organisations.^1^ ^46^ Gollop *et al*^47^ have indicated that individualised and tailored influencing techniques, such as finding the right ‘hook’ when making the case for change, might be required to reduce some healthcare professionals' scepticism and resistance to service improvement.

Embedded researchers can use their presence and daily working relationships to implement some of these ‘soft persuasive tools’. The researcher might facilitate meetings, provide technical assistance to solve problems and share their knowledge of the research evidence. They might tailor feedback by weaving in the host organisation's own words or letting actors from the host organisation take the lead.

Due to their knowledge of the organisation's context and culture, the researcher is able to share the findings in relation to the wider issues at stake in the organisation, such as the need to scale-up interventions or combine the study with wider improvement initiatives taking place across the organisation.^34^ ^37^ Furthermore, the researcher identifies and describes problems, and also cases of good practice,^34^ thereby helping to empower teams to continue with work that is producing positive outcomes.

### Capacity building

In many cases, embedded researchers help build research capacity so that the benefits of embedded research extend beyond the researcher's direct involvement. Capacity building might include promoting a reflexive culture before launching new initiatives, creating awareness of less well-known ways to approach problems, establishing a research culture, teaching evaluation skills or assisting in applications for external funding.^14^ ^34^ ^35^ In contrast to other research approaches that tend to be based on the development of individual partnerships between researchers and staff, the embedded approach centres on the incorporation of research into the organisation's systems, processes and practices, thus promoting its sustainability over time.^32^ ^35^

It is argued that embedded research also develops capacity at universities. It provides researchers with the opportunity to test methods and theories in practice—thus enhancing their applicability to real-life circumstances.^14^ ^23^ ^39^ Furthermore, the experience of working alongside healthcare professionals will help researchers later in their academic careers to design studies that generate insights helpful for healthcare organisations.^23^ ^33^ ^36^

### The challenges of carrying out embedded research

Carrying out rigorous research within healthcare organisations is challenging. For instance, some authors mention their hesitation when contemplating designing research that could potentially lead to negative results or highlight undesirable qualities of the host organisation.^34^ When attempting to disseminate findings, researchers might be bound by internal regulations that prevent them from publishing information considered harmful by the host organisation.^36^ The dual affiliation of many embedded researchers places them in a state of ‘in-between-ness,’ where they have to show their commitment to the organisation's goals and to the academic standards established for conducting publishable research in their fields.^37^ ^38^ This resembles the issues discussed in the literature on intermediary and boundary roles and the presence of role tensions or ‘role strain,’ a situation created when individuals have to deal with competing demands generated by members of the organisations they are affiliated to.^48^ ^49^

One way to deal with the challenges connected with dual affiliation is to agree on clear guidelines from the beginning to manage expectations.^50^ The guidelines might define the role of the researcher, types of studies they will be able to undertake, study timeframes and feedback processes.^14^ ^34^ Other embedded researchers have indicated that even if the researcher does not have an affiliation with an academic institution, it is important for him or her to maintain regular dialogue with academics about his or her research.^29^ ^37^ A connection with academia allows embedded researchers to keep up to date on new trends, preserve a critical perspective and make sure their research is rigorous.^37^ It has also been recommended that embedded researchers should foster relationships with other researchers doing similar work and share lessons of how to manage day-to-day issues.^29^

This review has highlighted a number of lessons that may be useful for embedded researchers, and these are summarised in box 2.

Box 2 **Lessons learned from the use of embedded researchers within and outside of the healthcare sector**

1. The embedded researcher needs to consider the experiences and points of view of the different subgroups within the organisation.
2. The creation of collaborative relationships with staff in the local organisation and the coproduction of knowledge can be enriched by practicing ‘reflexivity’ (reflecting on own position as an individual and as part of the organisational context).
3. Clear guidelines should be agreed between the embedded researcher and the local organisation from the beginning to manage expectations.
4. The researcher should schedule regular meeting with clinical teams and management groups to provide iterative feedback.
5. It is important for embedded researchers to maintain links with academic institutions to preserve a critical perspective.

### Limitations of this review

This review has a number of limitations. There is a lack of consensus around the terminology used to refer to embedded research. Our search terms and screening process might not have captured all of the relevant articles.

## Conclusion

Embedded research has the potential to address some of the main challenges in using research to improve quality in healthcare: understanding organisational culture to focus research appropriately, securing engagement from staff at different levels of the organisation to ensure the findings of research are translated into changes in practice and promoting the sustainability of improvement interventions.^1^ As Gold has argued, “in organisations, take up of ideas often occurs through informal processes”.^51^ The continuous presence of embedded researchers in healthcare organisations allows for the creation of these informal processes. The coproduction of knowledge between embedded researchers and local teams can lead to greater ‘ownership’ of the research findings by the healthcare organisation, and consequently, could lead to a smoother incorporation into changes in practice. The development of the research skills of local teams can help ensure research is viewed favourably and used within the organisation even after the embedded researchers have left.

Embedded research involves its own challenges in terms of dual affiliation, relationship building and sharing of results. Given our focus on the embedded research model as a method of coproducing research knowledge, we believe that maintaining a dual affiliation with health and academic organisations will represent a core component of embedded research. Learning from the experience of embedded researchers in different organisational contexts, and from the organisations they work with, could contribute further to our understanding of this approach. Rigorous evaluation of embedded research initiatives is required, which includes assessing the costs and benefits of embedded research for healthcare organisations.
