# Supplementary material for: The role of embedded research in quality improvement: a narrative review
Source: BMJ Qual Saf. 2016 Apr 29;26(1):70–80. doi: 10.1136/bmjqs-2015-004877 (PMC5256405; doi:10.1136/bmjqs-2015-004877)
Supplement: Supplementary appendix [file bmjqs-2015-004877supp_Appendix.pdf]

Appendix 1: Categories used in RedCap data extraction form

Categories used in RedCap data extraction form
Lead author
Country
Year
Sector
Type of article
Definition of embedded research
Characteristics of embedded research/researcher
Benefits of the embedded researcher role
Limitations of the embedded researcher role
Challenges of embedded research
Strategies to deal with challenges
Lessons learned
